# Risk factor analysis and idiographic features of mandibular coronoid fractures: A retrospective case–control study

**DOI:** 10.1038/s41598-017-02335-6

**Published:** 2017-05-19

**Authors:** Hai-Hua Zhou, Kun Lv, Rong-Tao Yang, Zhi Li, Zu-Bing Li

**Affiliations:** 1 The State Key Laboratory Breeding Base of Basic Science of Stomatology (Hubei-MOST) & Key Laboratory of Oral Biomedicine Ministry of Education, School & Hospital of Stomatology, Wuhan University, Wuhan, Hubei People’s Republic of China; 2 Department of Oral and Maxillofacial Surgery, College and Hospital of Stomatology, Wuhan University, Wuhan, Hubei People’s Republic of China

## Abstract

This study aimed to identify and distinguish various factors that may influence the occurrence of mandibular coronoid fractures. From January 2000 to December 2009, a total of 1131 patients with maxillofacial fractures were enrolled in this statistical study to evaluate the association between mandibular coronoid fractures and other risk factors. Among these patients, 869 had mandibular fractures, and 25 sustained a total of 25 coronoid fractures. More than half (13 of 25 patients, 52%) of the coronoid fractures in these patients were caused by motor vehicle accidents. Among these coronoid fractures, seven were associated with other mandibular fractures, and 23 (92.0%) were related to midfacial fractures. The most common site of midfacial fracture was the zygomatic arch (20 patients, 80%). Multivariate logistic regression analysis revealed that the most important influencing factor was the zygomatic arch fracture (odds ratio, 9.033; 95% confidence interval, 1.658, 49.218; *p* = 0.011). The majority of coronoid fracture fragments (19 of 25, 76%) were removed during operation. The most commonly used incision is hemicoronal or bicoronal approach (16 of 19, 84.2%).

## Introduction

Being the only mobile bone of the facial skeleton, the mandible is vulnerable to fracture because of its mechanically weak components, including the condyle, the angle, and both sides of the mentum; the mandibular fracture incidence rate is 23.8–81.3% in patients with maxillofacial fractures^1^. Although the coronoid process is a relatively weak part of the mandible, this area is rarely fractured due to its protected position deep under the zygomatic complex and the muscles that cover it^2^, making its fracture incidence rate accounting only to 1.23–3.58% of all mandibular fractures^2–5^.

Despite its low incidence rate, mandibular coronoid process fracture tends to result in serious complications, such as long-term pain and limited mouth opening (or truisms)^4^, Jacob’s disease^6^ and temporomandibular joint ankylosis^7^. The lack of consistency in the classification of mandibular coronoid process fracture pattern^5, 8, 9^, poses a controversy or divergence of opinion associated with coronoid fracture treatment among surgeons and researchers^2, 3, 10–13^. To date, few studies on mandibular coronoid process fractures have been conducted. A publication review showed a paucity of high-quality scientific data on the relationship between coronoid fractures and other influencing factors.

Findings on the investigation of the occurrence and patterns of mandibular coronoid fractures and the evaluation of the relationship between coronoid fractures and other influencing factors will provide us a comprehensive understanding of the epidemiological characteristics of mandibular coronoid fractures and guide to program design geared towards the prevention and treatment of those injuries. In the present retrospective case–control study, we aimed to analyse the aetiology, clinical symptoms and treatment of mandibular coronoid process fractures and evaluate various factors that may influence these fractures. The research data shows in detail the idiographic characteristic features of coronoid fractures. The aetiology, clinical symptoms and treatment of coronoid fractures are significantly different from those of other mandibular fractures.

## Materials and Methods

### Ethics Statement

We conducted a hospital-based retrospective case–control study at Stomatology College and Hospital, Wuhan University, from January 2000 to December 2009. The protocol as well as survey and consent forms were approved by the Institutional Review Board (IRB) of Wuhan University. Written consents provided by the patients were waived by the approving IRB.

### Patient Population and Data Collection

This study included patients with maxillofacial fractures admitted in the Department of Oral and Maxillofacial Surgery, Stomatology College and Hospital, Wuhan University, from January 2000 to December 2009. Patients with repeated admissions and incomplete information were excluded from this study. In total, 1131 participants with maxillofacial fractures had complete diagnostic records. Data on age, sex, soft tissue injuries, dental trauma and maxillofacial fracture type were collected and standardised by an investigator based on the patients’ case histories, clinical and radiographic examinations and medical records.

The injury mechanisms were classified as assault, road traffic accident (motor vehicle accident (MVA), motorcycle accident and bicycle accident), fall (at ground or high levels), sports- or work-related accident and others.

Midfacial fractures were categorised as zygomatic arch, zygomatic complex, orbital, maxilla, and combined zygomatic complex and arch fractures. Mandibular fractures were classified as condylar, symphysis, body, angle, ramus, and coronoid fractures.

Clinical symptoms included limited mouth opening, zygomatico facial depression, occlusal disorders, mouth opening deflection and temporomandibular joints tenderness.

Soft tissue and/or dental injuries in the maxillofacial area were recorded. Associated fractures, such as skull, thoracic, cervical, vertebra, pelvis, extremity, and abdominal injuries, were also documented as ‘other body fractures/injuries’.

### Case and Control Groups

Among the 1131 patients, those diagnosed with mandibular coronoid fractures comprised the case group. Meanwhile, patients with maxillofacial fractures but without mandibular coronoid fractures composed the control group.

### Statistical Analysis

Statistical analysis was performed using SPSS software (version 19.0; SPSS, Chicago, IL, USA). Continuous variables were reported as mean ± SD and were assessed using independent sample t-tests as necessary. Chi-square test was used to compare categorical variables. Fisher’s exact test was utilised when observation in any cell of the 2 × 2 table was expected to be less than five. Odds ratio (OR) and 95% confidence interval (CI) were used to assess the risk of sustaining mandibular coronoid fractures. Logistic regression analysis was utilised to control confounding variables. Probabilities of *P* < 0.05 were considered significantly different.

## Results

Based on the 10-year records retrieved during this study, 1131 patients were found to have sustained maxillofacial fractures. Of these patients, 869 had mandibular fractures, and 25 sustained a total of 25 coronoid fractures (accounting for 2.21% of patients with maxillofacial fractures and 2.88% of those with mandibular fractures). Among the patients with coronoid fractures, 21 were male and four were female with a male/female ratio of 5.25:1; both of these sexes have unilateral fractures. The age of patients with coronoid fractures ranged from 17 to 56 years old, with a mean of 35.00 ± 11.08 years. Among the 25 coronoid fractures, 14 were sustained on the left side, whereas 11 were sustained on the right side.

More than half (13 of 25 patients, 52%) of the coronoid fractures in these patients were caused by MVAs, followed by motorcycle accidents (4/25, 16%), assault-related accidents (3/25, 12%), fall at the ground level (2/25, 8%) and fall from a height (1/25, 4%).

Among the coronoid fractures, seven were associated with other mandibular fractures, three were linked to condylar fractures (1 bilateral, 3 unilateral/contralateral), another three were related to angle fractures (unilateral/contralateral), two were associated with symphysis fractures, one was linked to mandibular body fractures, and another one was related to alveolar fractures (Table 1).

**Table 1:** Characteristics of the patients sustained with mandibular coronoid fractures.

Patients	Sex	Age (years)	Coronoid fractures (Left/Right)	Etiology	Clinical symptoms^a^	Associated fractures in mandible (Left/Right)	Associated fractures in mid-facial^b^	Dental injuries	Soft tissue injuries in the maxillofacial^c^	Other body fractures/injuries	Treatment	Surgical approach
1	Female	42	Right	Assault	LMO; ZD(R); OD; PTMJA		R: ZCF, arch, maxilla, nasal		PL(L)		Removed	Hemi-coronal
2	Male	41	Right	Fall ground	MOD		R: Arch, ZCF, orbital, alveolar, maxilla	Yes	MS(R)/PL(R)/ML(L)		Removed	Hemi-coronal
3	Male	22	Right	Motorcycle	OD; TMJT(B)		R: ZCF		MS(R)		Conservative	Hemi-coronal
4	Male	30	Right	MVA	LMO; ZD(R); MOD		R: ZCF, arch		MS(R)		Removed	Hemi-coronal
5	Male	17	Right	MVA	LMO; ZD(R); OD		R:ZCF,arch, orbital, alveolar		PE(R)		Removed	Hemi-coronal
6	Male	33	Right	Fall high	LMO; ZD(B); OD		R: ZCF, arch, orbital, maxilla; L: ZCF, arch, maxilla	Yes	RVA(R)/EB(L)/PL(R)	Extremity (Upper right)	Conservative	Bi-coronal
7	Male	27	Right	MVA	LMO; ZD(R); MOD		R: ZCF, arch, maxilla; L: maxilla		MC(R)		Removed	Hemi-coronal
8	Female	55	Right	MVA	LMO; ZD(R)		R: ZCF, orbital, arch				Declined treatment	
9	Male	24	Right	Other	LMO; OD	Body (R)	R: Maxillary tuberosity		MS(R)/ML(R)		Removed	Submandibular
10	Female	46	Right	Other	LMO; OD; MOD	Angle (L),alveolar (L)	R: ZCF, arch; L: ZCF			Thoracic	Conservative	Tragus;Submandibular
11	Male	49	Right	MVA	LMO; OD; MOP; TMJT(B)	Condyle (L),condyle(R), sym		Yes	FL/SE		Removed	Tragus
12	Female	39	Left	MVA	LMO; ZD(L)		L: Arch		MS(L)/FN(L)		Removed	Hemi-coronal
13	Male	43	Left	Motorcycle	LMO; ZD(L); OD		L: Arch, orbital	Yes	PS(R)		Removed	Hemi-coronal; Intraoral approach
14	Male	37	Left	MVA	LMO; ZD(L)/TMJT(L)		L: ZCF, arch	Yes	PE(L)		Removed	Hemi-coronal
15	Male	56	Left	Assault	LMO; ZD(L)		L: ZCF, arch		ME(L)		Conservative	Hemi-coronal
16	Male	37	Left	MVA	LMO; ZD(L)		L: ZCF, arch				Removed	Hemi-coronal
17	Male	22	Left	MVA	LMO; ZD(L)		L: ZCF, arch, maxilla, Sphenoid, temporal bone		MS(L)	Skull	Removed	Hemi-coronal
18	Male	27	Left	Motorcycle	LMO; ZD(L); OD; MOD		L: ZCF, arch, orbital				Removed	Hemi-coronal
19	Male	21	Left	MVA	LMO; MOD		L: ZCF, arch, orbital		PL(L)/PN(L)	Extremity (Lower right)	Removed	Hemi-coronal
20	Male	35	Left	MVA	LMO; OD		L: ZCF, maxilla; nasal		PS(L)/PL(L)/IEC(R)/MOD	Skull, thoracic	Conservative	Hemi-coronal
21	Male	34	Left	Fall ground	LMO						Removed	Submandibular
22	Male	21	Left	Motorcycle	LMO	Angle(R)	L: arch, orbital		MS(L)/PS(L)/MT(L)	Extremity (Upper right)	Removed	Bi-coronal
23	Male	48	Left	Assault	LMO	Condyle(L)	L: ZCF, arch, maxilla, orbital; frontal, lateral pterygoid plate, internal pterygoid plate, sphenoid bone; R: Maxilla, orbital, lateral pterygoid plate, internal pterygoid plate, sphenoid bone; nose	Yes	MC(L)	Skull,extremity (Upper right; upper left)	Removed	Bi-coronal
24	Male	42	Left	MVA	LMO	Condyle(L)	L: Arch	Yes			Removed	Hemi-coronal
25	Male	27	Left	MVA	LMO; OD; od(L)	Sym(L), angle(R)	L: ZCF, arch	Yes	MS(L)/ME(L)	Extremeity(Upper left)	Removed	Hemi-coronal

Abbreviation: MVA: motor vehicle accidents; Right: R; Left: L; ^a^Clinical symptoms: Limited mouth opening: LMO; Zygomaticofacial depression; ZD; Occlusal disorders: OD; Mouth opening deflection: MOD; TMJ tenderness: TMJT; Bilateral: B; Poor TMJ activity: PTMJA; Mouth opening pain: MOP; Orbital depression: od; ^b^Associated fractures in mid-facial: ZCF: Zygomatic complex fractures; ^c^Soft tissue injuries in the maxillofacial: Maxillofacial swelling: MS; Maxillofacial laceration: ML; Periorbital laceration: PL;Periorbital ecchymosis: PE; Reduced visual acuity: RVA; eye blindness: EB; Maxillofacial contusion: MC; Forehead laceration: FL; Submandibular ecchymosis: SE; Facial numbness: FN; Periorbital swelling: PS; Maxillofacial ecchymosis: ME; Paranasal numbness: PN; Incomplete eye closure: IEC; Mouth opening deflection: MOD; Maxillofacial tenderness: MT.

Twenty-three patients (92.0%) were diagnosed with concomitant midfacial fractures. The most common site of midfacial fracture in patients with coronoid fractures was the zygomatic arch (20 patients, 80%), followed by the zygomatic complex (18 patients, 72%). Sixteen patients (64%) with coronoid fractures have concomitant zygomatic complex and arch fractures. Other midfacial fracture-associated sites included the orbit (9 patients, 36%) and maxilla (8 patients, 32%). Additionally, nearly all midfacial fractures in patients with coronoid fractures were ipsilateral. Only one patient without any mandible or midfacial skeleton fracture was recorded (Table 1).

Eight of the coronoid fractures (32%) were associated with dental injuries (data not listed in Tables), and another eight (32%) were related to body injuries. Nineteen (76%) of the coronoid fractures were removed during operation. Five coronoid (20%) fractures were treated via conservative therapy. One patient with coronoid fractures declined treatment because of diabetes. None of these patients were treated via open reduction and internal fixation. Among the patients with coronoid fragments removed during the operation, 16 underwent semi-circular or coronal incision, two were treated through submandibular approach, and one was treated via the tragus approach. The patient having only coronoid fracture (with neither mandibular nor midfacial fracture) was treated through submandibular incision (Table 1).

Almost all aetiologies seemed to display low risk of mandibular coronoid fractures (OR < 1), except for MVAs (OR > 1) (Table 2). However, statistical analysis showed that no significant relationship existed between the different aetiologies and coronoid fractures. Patients with midfacial fractures showed high risk (OR > 1) to coronoid fractures, especially those with zygomatic arch fractures (OR, 13.696; 95% CI, 5.089–36.861; *p* < 0.001) or zygomatic complex fractures (OR, 7.408; 95% CI, 3.062–17.919; *p* < 0.001) or those with concomitant zygomatic complex and arch fractures (OR, 9.026; 95% CI, 3.928–20.740; *p* < 0.001). Patients who have fractures on other parts of the mandible had lower risk of coronoid fractures (OR, 0.121; 95% CI, 0.050, 0.292; *p* < 0.001). However, the result of multivariate logistic regression analysis confirmed that the most important factor is the zygomatic arch fracture (OR, 9.033; 95% CI, 1.658, 49.218; *p* = 0.011) (Table 3).

**Table 2:** Multivariate logistic regression: risk of mandibular coronoid fractures in patients by age, gender and etiology.

Factors	Case (n = 25)	Control (n = 1106)	Crude		Adjusted	
			OR (95%CI)	*p*	OR (95%CI)	*p*
Sex			1.502 (0.511, 4.416)	0.457	1.397 (0.470, 4.148)	0.547
Male	21	860				
Female	4	246				
Age	35.00 ± 11.08	31.00 ± 13.48	—	0.141	0.980 (0.951, 1.010)	0.180
**Etiologies**						
Assault	3	156	0.830 (0.246, 2.807)	1.000	0.658 (0.107, 4.036)	0.651
Bicycle	0	67	—	0.394	—	0.997
MVA	13	336	2.483 (1.121, 5.498)	0.021	1.449 (0.319, 6.593)	0.631
Fall ground	2	134	0.631 (0.147, 2.706)	0.759	0.571 (0.078, 4.166)	0.581
Fall high	1	120	0.342 (0.046, 2.553)	0.507	0.323 (0.029, 3.647)	0.361
Motor	4	175	1.013 (0.344, 2.988)	1.000	0.880 (0.156, 4.960)	0.885
Other	2	71	1.268 (0.293, 5.484)	0.673	NA	NA
Sport	0	20	—	1.000	—	0.998
Work	0	27	—	1.000	—	0.998

NA: Not application.

**Table 3:** Multivariate logistic regression analysis: risk of mandibular coronoid fractures in patients by fracture of mid-facial or other part of mandible, dental injuries, and maxillofacial soft injuries.

Factors	Case (n = 25)	Control (n = 1106)	Crude		Adjusted	
			OR (95%CI)	*p*	OR (95%CI)	*p*
ZCF fractures	18	285	7.408 (3.062, 17.919)	<0.001	2.644 (0.364, 19.181)	0.336
Arch fractures	20	250	13.696 (5.089, 36.861)	<0.001	9.033 (1.658, 49.218)	0.011
Orbital fractures	9	125	4.414 (1.910, 10.201)	0.001	1.307 (0.518, 3.300)	0.571
Maxilla fractures	7	123	3.108 (1.273, 7.591)	0.018	1.239 (0.462, 3.320)	0.670
ZCF + arch fractures	16	182	9.026 (3.928, 20.740)	<0.001	0.492 (0.053, 4.570)	0.533
Other mandible fractures	7	844	0.121 (0.050, 0.292)	<0.001	0.463 (0.154, 1.391)	0.170
Dental injuries	8	465	0.649 (0.278, 1.516)	0.314	1.333 (0.521, 3.412)	0.549
Maxillofacial soft injuries	19	842	0.993 (0.392, 2.512)	0.988	0.862 (0.327, 2.274)	0.764

Table 4 lists the distribution of coronoid fracture treatment methods based on various factors. The data analysis showed that no statistically significant association existed between the coronoid fracture treatment methods and various factors.

**Table 4:** Distribution of treatment methods of coronoid fractures according to various factors.

	Surgery (n = 19)	Non-surgery (n = 5)	*p*
Age (years)	33.1 ± 9.8	38.4 ± 13.0	0.321
Sex (male)	17	4	0.521
MVA-related	11	1	0.317
LMO (cm)	1.3 ± 0.8	0.8 ± 0.6	0.217
Soft tissue injuries	15	4	1.000
Dental injuries	7	1	0.631
ZCF	12	5	0.272
Arch fracture	16	3	0.270
ZCF + Arch fracture	12	3	1.000
Open treatment of ZCF/Arch fractures	16	4	1.000
Hemi or bi-coronal approach	16	4	1.000

## Discussion

Despite the large number of articles on maxillofacial fractures, few studies have been conducted specially on the epidemiological characteristics of coronoid fractures. The research data of the present retrospective case–control study shows in detail the idiographic characteristic features of coronoid fractures. The aetiology, clinical symptoms and treatment of mandibular coronoid fractures are significantly different from those of other mandibular fractures^1, 14–17^.

Coronoid fractures account to 1.23–3.58% of all mandibular fractures^2, 4, 5^ and 0.85–2.9% of all maxillofacial fractures^3, 4, 7^. In this study, the overall prevalence of coronoid fractures associated with mandibular fractures was 2.88%. This figure is highly close to that obtained by Shen *et al*.^5^ (2.90%), higher than that determined by Singh *et al*.^4^ (1.23%) and lower than that acquired by Kale *et al*.^2^ (3.58%). The overall prevalence of coronoid fractures associated with maxillofacial fractures was 2.21%, which is higher than that obtained by Boffano *et al*.^7^ (1.16%) and Singh *et al*.^4^ (0.85%) and lower than that determined by Rapidis *et al*.^3^ (2.9%).

Studies on the epidemiological characteristics of coronoid fractures in a large sample showed that different countries have various trauma aetiology patterns. Violence is highly related to coronoid fractures in South Africa (86.67%)^4^. Road traffic accidents is the most common cause of fractures in Greece^3^, India^2^, China^5^ and two European countries (the Netherlands and Italy)^7^. Our studies confirmed the results of the investigations by Rapidis *et al*.^3^, Kale *et al*.^2^, Shen *et al*.^5^ and Boffano *et al*.^7^ (*p* < 0.001; data was not listed). Additionally, several case reports are also available on iatrogenic fractures occurring during maxillary and mandibular third molar extractions, cystectomies and sagittal split ramus osteotomies^18, 19^. Interestingly, no significant relationship existed between the different aetiologies and coronoid fractures, which are highly distinguished from other mandibular fractures. Previous studies revealed that mandibular fractures are significantly related to traumatic aetiologies^14, 15^.

Diagnosing coronoid process fractures only by clinical symptoms is very difficult. In the present study, almost all patients (23 of 25 patients, 92%) with coronoid fractures showed limited mouth opening, more than half (13 of 25 patients, 52%) experienced zygomaticofacial depression, and nearly half suffered from occlusal disorders (11 of 25 patients, 44%). It is worth mentioning that 80% and 72% of our patients have associated zygomatic arch fractures and zygomatic complex fractures, respectively. Actually, in most cases, coronoid fractures coexist with other mandible or midfacial fractures; the signs and symptoms of the fractures from other sites predominate the clinical symptoms^3^. Considering the above findings, spiral computed tomography and panoramic radiographs are still the gold standard (or extremely valuable) procedures in the diagnosis of mandibular coronoid fractures^20^.

The mechanism underlying coronoid fracture is unclear. Until now, the zygomatic arch (or zygomatic complex) is generally considered as a shield to the coronoid process^9, 12, 18, 21–24^. Coronoid fracture is highly distinguished from other mandibular fractures in that internal interactions between the different mandibular fracture sites exist^1^; even dental trauma is highly related to mandibular fractures^16, 25^. If the zygomatic arch can really protect the coronoid process, deducing that zygomatic arch or complex fracture can significantly reduce the risk of coronoid process fracture development is reasonable. However, many studies had reported that coronoid fractures are usually associated with zygomatic arch or complex fractures^3, 7, 13, 24, 26^. In the present study, we further revealed that patients with zygomatic arch fractures showed the highest risk (OR, 9.033; 95% CI, 1.658, 49.218; *p* = 0.011) to coronoid fractures. Nonetheless, at present, no direct evidence indicating that zygomatic arch fracture leads to secondary coronoid process fracture (squeeze injury) is available, despite their adjacent relationship in space. One study examined 15 patients with coronoid fractures associated with only one zygomatic arch fractures^4^. Some scholars are in favour of the theory that acute temporalis contraction leads to coronoid process fracture^4, 24, 26–28^. The temporalis muscle, which is large and has a fan shape and arises from a broad base and inserts into the medial aspect and tip of the coronoid process, helps in mandibular elevation and retrusion^26^. Coronoid process fracture is usually caused by a blow to the temporal region after an assault or collision. This fact is the reason why Singh *et al*.^4^ reported only one patient who has associated zygomatic arch fractures with no evidence of direct trauma to the facial bones. The plausible reason for the coronoid fracture is acute reflex contraction of the temporalis muscles after an assault. Therefore, zygomatic arch fracture (or depression) resulting in impact on or collision with the temporalis muscle seems to lead to acute temporalis contraction and, consequently, to coronoid process injury and even coronoid fracture. From the research standpoint, using synthetic bone (or similar products) in the lab to determine if certain impacts from a given vectoral direction and magnitude can recreate the same fracture pattern would be necessary^1^.

Several mandibular coronoid fracture classifications have been used in the literature. Natvig *et al*.^8^ categorised coronoid process fractures into two types: intramuscular and submuscular. The distinction between these types was based on whether the coronoid fracture fragment is within the temporalis attachment or not. Considering this classification, scholars^8, 11^ believed that intramuscular fractures do not require operative treatment because the muscular spasm of the temporalis is usually sufficient to hold the fragment in position until healing, whereas displaced submuscular fracture may be treated via occluded teeth fixation^8^ or open reduction and intraosseous wiring^10^. Shen *et al*.^5^ divided coronoid fractures into linear fracture (further classified into coronoid base, upper coronoid process and combined coronoid process and mandibular ramus fractures) and comminuted fracture. These authors advised that fresh, linear coronoid fractures with minimal displacement or clinical symptoms can be managed conservatively, whereas rigid internal fixation is recommended for fractures with significant displacement, limited mouth opening and concomitant mid-face or mandibular fractures, especially if osseous union between the coronoid process and zygomatic arch is present. Brener and Alley^9^ classified coronoid fractures as transverse and longitudinal fractures. Regardless of the classification, treatment eventually depends on the degree of coronoid fracture displacement and symptom severity^2, 3^; the therapy is aimed at ankylosis prevention and early mobilisation of the mandible^7, 20^. Some authors recommended removal of the coronoid process in cases with fibrous union between the zygomatic arch and coronoid process^11^ or those with movement limitation due to temporalis muscle fibrosis^12^. In a review, Kisnisci^13^ also underlined a commonly applied surgical technique in fractured segment removal and immediate postoperative exercises. In the present study, 19 patients (72%) underwent coronoid fracture removal during operation, whereas only five patients with coronoid fractures (20%) were treated using conservative therapy. One patient with coronoid fractures declined treatment because of diabetes. None of these patients were treated via open reduction and internal fixation. In our experience, the coronoid fracture treatment was dependent on whether or not the limited mouth opening is relieved during the treatment procedure of other maxillofacial fractures. Coronoid fracture removal is carried out if limited mouth opening cannot be resolved after open reduction of maxillofacial fractures. This observation can also explain why the treatment of coronoid fractures is independent of other external factors in the present study (age, sex, aetiology, mouth opening after injury, associated injuries, surgical approaches, etc.) (Table 4).

Surgical treatments for coronoid fractures are classified into intraoral, external (submandibular/hemicoronal, bicoronal, etc.) or a combination of both. The intraoral approach avoids skin scar and eliminates facial nerve damage risk, but occasional buccal pad herniation into the surgical site can occur^29^. The use of this approach is also limited in cases of severe trismus^30^. The main disadvantages of the external approaches include poor aesthetics and facial nerve branch damage. Nonetheless, the coronal approach provides better visualisation (or excellent access)^30, 31^ and an acceptable scar behind the hairline^30^, especially for patients with associated zygomatic arch or complex fractures. In the present study, among the patients treated via operation, 16 underwent semi-circular or coronal incision, two were treated via the submandibular approach, and one was treated through the tragus approach. None of these patients were treated via open reduction and internal fixation. The treatment of coronoid fractures in our department is also different from that of other mandibular site fractures^14, 17^.

We acknowledge the considerable difference in the number of samples between the case and control groups in this retrospective study. However, the present study provided a good model in the evaluation of various factors that may influence the coronoid fractures. Furthermore, the results of this study will be particularly helpful in confirming the findings of a case–control study in patients with rare disease. Nonetheless, a prospective, multicentre and large sample case–control study should be conducted in the future, through which the relationship between coronoid fracture and other factors can be evaluated more thoroughly and accurately.

In conclusion, coronoid fracture occurrence is rare and significantly related to zygomatic arch fracture. Most of the coronoid fractures were caused by road traffic accidents. Furthermore, the majority of the coronoid fracture fragments were removed during operation. The most commonly used incision is hemicoronal or bicoronal approach. The aetiology, clinical symptoms and treatment of mandibular coronoid fractures are significantly different from those of other mandibular fractures.
